# Health, Psycho-Social Factors, and Ageism in Older Adults in Spain during the COVID-19 Pandemic

**DOI:** 10.3390/healthcare9030256

**Published:** 2021-03-01

**Authors:** Rocío Fernández-Ballesteros, Macarena Sánchez-Izquierdo

**Affiliations:** 1 Department of Psychobiology and Health, Autonomous University of Madrid, 28049 Madrid, Spain; r.fballesteros@uam.es; 2 Department of Psychology, Universidad Pontificia Comillas, 28049 Madrid, Spain

**Keywords:** health, ageism, COVID-19, pandemic, older adults, ageism

## Abstract

Older adults are a population at risk for COVID-19. This study has two independent objectives: (1) to report the impact of COVID-19, as well as psycho-social responses during lockdown, in a sample of older adults in Spain, and (2) to explore through the review of published surveys what extent ageism has increased at the population level. The first objective was to search through an online questionnaire collecting information about self-reported health, lifestyles, psycho-social conditions, and a diversity of concerns. This questionnaire was administered to a volunteer sample of 315 older Spanish men and women (301 COVID-19-free and 14 diagnosed with COVID-19). All individuals reported that they had information about the COVID-19 pandemic. Their self-perception of health was also quite good, most maintaining healthy lifestyles and few reporting unhealthy behaviors. They reported few changes in family and interpersonal relations during lockdown. Those diagnosed with COVID-19 reported higher levels of anxiety, irritation, and fear, in comparison with the COVID-19-free group. Interestingly, instead of being concerned about health, the greatest concern in both groups (COVID-free and those diagnosed with COVID) were politics and the future. Our second objective, to explore ageism during the first wave of the COVID-19 pandemic, was examined across various surveys conducted in several populations by several authors. Results showed an increase in ageism in Spain. Although some new information about health perception, psychosocial responses, and concerns during this unknown stressful situation was obtained, much more research with representative samples is required in order to arrive at more accurate conclusions.

## 1. Introduction

In Spain, the first COVID-19 patient was diagnosed in La Gomera, Canary Islands, on 31 January 2020—one month after the Wuhan epidemic was declared. On 2 March, 119 COVID-19 cases were reported by the National Epidemiology Centre, of which 79% were men and 27% were older than 65 years. Ten days later, the situation was out of control, the Spanish Government declared a state of alarm, and the whole country was placed in lockdown. Two months later, on 9 May [1], Spain reported 231,765 COVID-19 cases and 18,342 deaths, with 35.2% aged over 70 years and a very high percentage of deaths in senior residences (lethality by age range: 70–79 = 12.7 and ≥80 = 26.28, higher than those reported by Sánchez-Rodríguez [2] (of 70–79 = 8% >80 = 14.8; see update [3]). Throughout the pandemic, several authors have emphasized the emerging threat of ageism—as has occurred in other similar pandemics in the past—and the literature throughout 2020 has tested diverse sources of data including international contexts [4,5].

COVID-19 is defined as a new type of coronavirus that spreads rapidly from person to person, resulting in a major epidemic. Although this disease fits a bio-medical model, as Horton [6] underlined, the whole situation constitutes a synergy of bio-psycho-socio-environmental circumstances to which a *syndemic* model [7] is better suited. In other words, the bio-medical model must be complemented by other health approaches, as has been recently emphasized by several authors [8]. Furthermore, several studies carried out in Spain have shown a significant degree of psychological impact, anxiety, depression, loneliness, and stress, not only among frontline workers [9] but also among the general population [10,11,12,13]. Thus, the bio-medical model must be complemented by other health perspectives, as has recently been emphasized [8], taking into consideration potential multidisciplinary risk and protective factors, including psycho-social ones. Little is known about how older adults are coping with this situation, although several previous studies have found that older adults show better emotional well-being and less reactivity to COVID stressors than younger adults [14].

Our objectives are the following: (1) To reveal both risk and protective behavioral factors, as antecedents of COVID-19, as well as personal experience around this illness; (2) to detect individual responses to the new circumstances due to the pandemic situation (as an unknown and broadly stressful situation, never experienced before) such as confinement, social distancing, and other physical and social constraints, and (3) to explore to what extent this situation is increasing ageism. In summary, the general purpose of the study is to learn how to combat and efficiently cope with this in similar situations in the future.

## 2. Materials and Methods

### 2.1. Participants

Members of the CEOMA (Confederation of Older Spanish Adult Associations) from 15 regions in Spain with 430,000 members, were invited to participate in this study. A volunteer sample of older adults living in the community (*N* = 315; error ± 5; confidence 92%) completed the survey.

### 2.2. Questionnaire

A questionnaire for data collection was administered through Google Forms (see: https://forms.gle/HxEqARdf8pYwJkxx7 (accessed on 5 May 2020)). The questionnaire contained 50 items, with the following sections: sociodemographics, COVID-19 risk, and protective factors and experiences; self-reported health; lifestyles; unhealthy behavioral patterns; feelings and emotions, coping and self-efficacy; family network and social support and satisfaction; and concerns experienced during this first wave of the pandemic.

Section one of the questionnaire comprised 10 questions on the socio-demographic characteristics of participants. Information such as age, gender, educational level, work, and marital status was obtained in this section. In section two, 17 questions related to lockdown adherence, being diagnosed with COVID and symptoms, care received and satisfaction with the care received, and knowledge about the disease and information sources. The third section had 13 questions related to the respondents’ perception of their health and healthy or unhealthy habits during lockdown. In section four, five questions were provided about family and interpersonal relations during lockdown and activities in which the person was involved during lockdown. The final section contained five questions about coping with the situation.

### 2.3. Procedure

The data were collected online through a Google Forms questionnaire. The data collection period was from 1 April to 5 May 2020. To recruit the sample, CEOMA (Confederation of Older Spanish Adults Associations) was contacted and asked to disseminate the study. All respondents provided informed consent prior to accessing the questionnaire.

### 2.4. Statistical Methods

Descriptive statistics were calculated for the sociodemographic characteristics of the sample and the study variables, consisting of frequencies and percentages for categorical variables and means and standard deviations (SDs) for scale variables. The statistical analyses were performed using IBM SPSS Statistics for Windows, version 19 (IBM Corp., Armonk, NY, USA).

## 3. Results

### 3.1. Sociodemographics

Table 1 shows the two subsamples, COVID-19-free (F-COVID) and infected by COVID-19 (I-COVID). Although COVID-19 prevalence at a similar age-range was approximately 20% at the population level, only 4.4% (*N* = 14) of our sample was COVID-19-infected (6 men and 8 women; age range = 66–77 years; 65%). Therefore I-COVID individuals were younger and with a higher proportion of women than our total sample. Nevertheless, no other significant sociodemographic differences were found among these two subsamples.

### 3.2. COVID19 Experiences and Protection and Risk Factors

Although COVID-19 is a virus described by a bio-medical model, from a health psychology point of view, the socio-behavioral protective and risk factors reported in the questionnaire must also be taken into consideration. We will start by describing differences in both protection and risk factors among the I-COVID and F-COVID subsamples.

1. Without a doubt, because information modulates behavior, a first protective factor is information about COVID19. The two subsamples reported obtaining information from a variety of sources, mainly newspapers, radio, or TV (91%/85.7%). However, a lower percentage reported information from scientific literature (48.8%/42.9%).
2. A second important protective factor is confinement or mandatory quarantine. A total of 42.5% in the F-COVID and 57% I-COVID declared that they followed these instructions. It must be emphasized, however, that 55% of F-COVID and 43% of I-COVID did not answer this section. In summary, in comparison with the F-COVID subsample, I-COVID participants had good information about COVID and declared that protective instructions were followed.
3. The COVID-19 virus is highly contagious through person-to-person contact, a fact addressed in the questionnaire. In the I-COVID sample, 13 out of 14 (92.9%) individuals reported that they had had contact with friends or a family member with COVID-19. In contrast, this was the case with only 9.8% in the F-COVID group.

In summary, although healthy and infected participants did not differ in terms of information and lockdown adherence, I-COVID individuals seem to have been infected through contact with other patients.

With respect to the treatment and care received by I-COVID individuals, it must be emphasized that five individuals did not reply to this section; therefore, we are referring only to nine I-COVID individuals: seven remained at home, receiving primary health care, and two were hospitalized, but none went into intensive care (ICU). Only three reported being tested with PCR because, at the time of the study, COVID-19 clinical diagnosis was through the standard primary care procedure since PCR kits were not yet available.

Regarding satisfaction with care, (answered by 12/14): ten I-COVID reported being “very satisfied” (three women and two men) or “rather satisfied” (two women and three men), one woman reported being “somewhat satisfied”, and only one woman reported being “not at all satisfied”. Complaints about care were the following: “no answer received”, “no test conducted”, “lack of follow-up of symptoms”, “lack of empathic interaction”, and “lack of information given due to deficient management by the government”. These open responses are very similar to those provided in the final section related to ageism during the pandemic.

In summary, although from a statistical point of view it must be concluded that a majority of I-COVID individuals were satisfied with the care received, some reported the diversity mainly in primary health care at the beginning of the pandemic when the health service had collapsed under the unforeseen situation.

### 3.3. Self-Reported Health during the Pandemic

The purpose here was to assess subjective health through self-reports during this first wave of the COVID-19 pandemic in Spain. Of the F-COVID cases reported, 91% had “good” (45.2%) or “very good” (47.8%) health. This is not significantly different, however, from the I-COVID group: 10 of 14 (six women and four men) rated their health as “very good”, and the other four (two men and two women) as “rather good”; none of the participants reported “bad” health. It is important to emphasize that, unfortunately, information about when the I-COVID individuals were infected was not collected.

When asked to compare themselves with other people of the same sex and age, the F-COVID group reported having the following: 6.6% “much better”, 44.9% “better”, 49.5% “same”, and only 5.6% “worse” health. Regarding I-COVID, half (three women and four men) reported “better” health, 35.7% “same” health, and only two people (14.3%) reported their health as “worse”. In summary, all reported good health after being infected by COVID-19.

Finally, comparing their current health with their health before the COVID-19 pandemic (Table 2), 70% of the F-COVID group reported that it was better (62% “somewhat better”, 6.6% “much better”), and only 16.2% “somewhat worse”. In the I-COVID group, half reported being “somewhat worse” but the other half rated themselves as “somewhat better”.

In summary, most participants in both groups, F-COVID and I-COVID, rated themselves as having good health, and very few of them as worse compared to others of the same age and gender. Only when they were asked to compare their health before and after the pandemic did differences among them appear; F-COVID self-evaluation was better than I-COVID.

### 3.4. Changes in Healthy Lifestyles

The extent to which the COVID-19 pandemic situation, including compulsory confinement and other legal restrictions in Spain, could necessitate changes in daily life activities, including healthy habits or lifestyles, is also an important health issue. The questionnaire thus contained items asking about how far healthy lifestyles were reduced, maintained, or increased.

A very similar pattern, depending more on the type of lifestyle than being or not being infected with COVID-19, is shown in Table 3. Thus, physical exercise, more as a result of external circumstances, was reduced in both groups, by 78.6% in I-COVID and 65.1% in the F-COVID group. On the other hand, three healthy habits, maintaining cognitive activity, following a healthy diet, and adherence to doctors’ prescriptions were maintained in both groups to very high proportions (ranging from 64.3% in I-COVID maintaining cognitive activity to 83.1% in the F-COVID getting their prescriptions filled). Finally, cognitive activity can be increased under certain physical constraints, such as lockdown.

In summary, while physical exercise was reduced, as expected, in both F-COVID and I-COVID, maintaining cognitive activity, following a healthy diet, and getting prescriptions filled were maintained in both.

### 3.5. Unhealthy Behaviours as a Response to the COVID Pandemic

Health information was also complemented with self-reports about unhealthy behaviors as a response to the pandemic. Thus, drinking alcohol, smoking tobacco or use of other drugs, overeating, or sedentarism could be responses to the pandemic. When differences among the two groups were compared, minor differences were found among behavioral patterns. Approximately half of both the I-COVID and F-COVID groups reported overeating (F-COVID = 61.5%; I-COVID = 64.3%) and alcohol consumption (F-COVID = 45.2%; I-COVID = 57.1%), but no differences were found between both groups. Minor percentages were found in sedentary behaviour (F-COVID = 23.6%; I-COVID = 23.6%), smoking (F-COVID = 13.3%; I-COVID = 14.3%), and drug use (F-COVID = 9% I-COVID = 8.1%). The frequency of these more frequent unhealthy behavior patterns was low (“somewhat frequent”) in both groups, and there were no significant differences between them.

It could be concluded that two unhealthy behaviors, specifically overeating and drinking alcohol, occurred in at least half of both F-COVID and I-COVID individuals as a response to the pandemic. Furthermore, associations were found between unhealthy behavior and changes in lifestyle.

### 3.6. Emotions and Their Repercussions in Daily Life

Since the COVID-19 pandemic must be considered a stressful situation, significant differences in negative emotions between F-COVID and I-COVID could be expected to be found as responses to being infected or not. Therefore, questions about self-reported emotions as well changes in sleeping patterns, considered as an indirect expression of stress, were introduced in the questionnaire (Table 4).

Regarding feelings of anxiety, 23.6% reported no anxiety at all in the F-COVID group, but 38.5%, 29.6%, and 8.3% reported feeling “somewhat“, “rather”, and “very” anxious, respectively. On the other hand, none of them reported “no anxiety”, 9 (64%) reported “somewhat”, and 4 (28.6%) reported feeling “rather” anxious in the I-COVID group.

Regarding depression, the I-COVID group reported lower levels of depression (all of them reported either no depression at all or being somewhat depressed), while 79% of individuals in the F-COVID group reported no depression or some level of depression, with 16.6% being “rather”, and 4.3% “very much” depressed.

Regarding irritation, nearly half (47.2%) of the F-COVID group did not report feelings of irritation at all, but half reported some degree: 30.9% “somewhat”, 16.9% “rather”, and 4.7% “very much”. Regarding the I-COVID group, a third did not report irritation at all but two-thirds reported “some” degree of irritation.

With respect to fear, the response distribution is quite similar in the two groups, with 26.6% of F-COVID and 21.4% of I-COVID not reporting fear at all, and “some” fear reported by 43.5%/28.6%, “rather afraid” by 20.6%/35.7%, and “very afraid” by 12%/14.3%.

However, when older adults were asked about the extent to which their emotions were involved in making their daily life difficult, both groups—F-COVID and I-COVID—seemed to maintain their functionality in spite of their emotions, with half of the F-COVID group (51.8%) and almost half of I-COVID (42.9%) answering “not at all”. This question was only rated “very much” by 3% in F-COVID and 7% in I-COVID.

Finally, an attempt was made to obtain some indirect information about their negative feelings and worries by asking about changes in their sleeping patterns. While 60% of the F-COVID group individuals did not report any changes, very few reported sleeping disturbances. In the I-COVID group, 35% reported changes in their sleeping pattern; however, there was high variability in this group—with five participants having slept as usual and nine reported sleeping problems during lockdown.

In summary, verbal reports of anxiety, irritation, and feelings of fear were higher in the I-COVID than in the F-COVID group, which was confirmed through more sleeping disturbance in the infected group compared to the F-COVID group.

### 3.7. Psychological Factors of COVID19: Perceived Severity, Coping, and Self-Efficacy

COVID-19-perceived severity, coping, and self-efficacy are important psychological factors. Thus, the questionnaire asked to what extent COVID-19 was considered a serious illness and the pandemic situation was experienced as difficult to cope with in comparison with other stressful situations during a person’s life and, finally, to what extent the situation was perceived as being solved with efficacy.

Regarding *the perception of illness severity*, 85.7% of F-COVID individuals perceived COVID-19 as “very serious” in comparison with 70.4% of those with I-COVID. Furthermore, 14.3% of F-COVID individuals perceived COVID-19 as less “serious” in comparison with 27.2% of individuals in the I-COVID group who perceived it only as “serious”. With respect to the difficulty of the situation in comparison with other stressful events in the past, significantly more F-COVID than I-COVID individuals perceived the pandemic situation as being difficult (76% of F-COVID individuals answered “rather” or “very high” difficulty in comparison with 57.2% of I-COVID individuals). Finally, regarding the extent to which they perceived themselves as self-efficacious in coping with the COVID-19 pandemic, 88% of F-COVID individuals and 93% of I-COVID individuals answered “rather” or “very much” coping efficacy.

In summary, more non-infected individuals perceived COVID-19 illness as being more serious and reported the pandemic as being a more difficult stressful situation than others in the past. At the same time, all considered themselves to be very close to family and highly self-efficacious in coping with the situation.

### 3.8. Family and Social Interactions and Satisfaction

COVID-19 as an illness and a pandemic has had important repercussions at the individual, family, and social levels. Therefore, the questionnaire covered a set of items asking about changes in those relationships during the first wave of the pandemic.

An overview of the differences in the family network of the F-COVID and I-COVID groups shows that 72.1% F-COVID were living with their partners, 7% with their children, 16.6% alone, and 3.7% with other family members or caregivers, while 57.1% of the I-COVID group were living with their partners, 21.4% with their children, and 21.4% alone. Furthermore, while 82.3% in the F-COVID group were “very much” or “rather satisfied” with those relationships, 92.8% I-COVID individuals were very much or rather satisfied with their family network.

Table 5 shows the reported *changes* occurring to participants in their interactions with the support received, and satisfaction (reduced, maintained, improved) with respect to significant others (partner, children and/or grandchildren, other family members, friends, and others) during lockdown.

Participants in both the F-COVID and I-COVID groups reported less than a 15% reduction in their interpersonal relations, and most of them maintained similar close interactions with significant relatives: 64.3% in the I-COVID group, 54.8% in members of the F-COVID group with their partner; 78.6% in I-COVID and 53.8% in F-COVID group with their children; 57.1% in the I-COVID group and 49.8% in the F-COVID group with other family members. Finally, 50% both in the I-COVID and F-COVID groups maintained close interactions with their respective friends. Moreover, most of the changes reported were improvements in their relationships: 21.4% in the I-COVID group as well as 18.3% in the F-COVID group with their spouse or partner; 14.3% in the I-COVID group and 32.6% in the F-COVID group with their children. Finally, 21.4% in the I-COVID group and 39% in the F-COVID group with other families as well as 42.9% in the I-COVID group and 41% in the F-COVID group with their respective friends.

With respect to *change in support received*, similar patterns of reduction were shown in both the F-COVID and I-COVID groups. Nevertheless, maintenance rather than change was the norm in both the I-COVID and F-COVID groups (57.1% in I-COVID and 50.2% in F-COVID with their partner; 64.3% in I-COVID and 44.2% in F-COVID with their children; 57.1% in I-COVID and 52.5% in F-COVID with other family members; 42.9% in I-COVID and 50.2% in F-COVID with their friends. Additionally, improvement in support received increased by a relatively high proportion in both the I-COVID and F-COVID groups: from their partners 28.6% in I-COVID and 23.3% in F-COVID; from their children 35.7% in I-COVID and 43.9% in F-COVID; from their family members 42.9% in I-COVID and 36.9% in F-COVID, and from their friends 57.1% in I-COVID 42.5% and in F-COVID, from their respective friends.

Regarding *satisfaction* with respect to the support received, both groups reported very similar satisfaction levels with all network members. With partners, I-COVID reported feeling “rather” 28.6% or ”very” 35.7% satisfied and F-COVID reported “rather” 22.3% or “very” 39.2% satisfied. With children, I-COVID reported feeling “rather” 35.7% or “very” 57.7% satisfied and F-COVID reported “rather” 27.2% or “very” 50.2% satisfied. With family members, I-COVID reported feeling ”rather” 42.9% or “very” 35.7% satisfied, and F-COVID reported “rather” 38.9% and “very” 33.2% satisfied. With friends, I-COVID reported feeling “rather” 42.9% and “very” 35.7% satisfied, while F-COVID reported “rather” 40.2% and “very” 35.5% satisfied. With social networks, I-COVID reported feeling “rather” 35.7% or “very” 14.3% satisfied, while F-COVID reported “rather” 28.9% and “very” 11.3% satisfied. Finally, I-COVID reported 21.4%, and F-COVID 52.3% satisfaction with other network members. The highest source of satisfaction received was from children in both groups; 57% of I-COVID participants and 50% in the F-COVID group reported “very much” satisfaction. Finally, very few individuals in both groups reported “no or little” satisfaction with any of the network members.

In summary, both I-COVID and F-COVID participants reported very few changes in their relationships during lockdown, and some of them improved their relationships. Regarding the support received and feelings of satisfaction, the pattern of change is quite similar, most reporting no changes and even some improvements. Finally, the highest source of improvement in support and satisfaction were children and friends.

### 3.9. Concerns about Health, Work, the Economy, Politics, and the Future

The COVID-19 pandemic not only refers to the disease but has also brought about a strong socio-economic crisis, with personal, family, and social repercussions. The questionnaire, therefore, contained questions about the extent to which individuals were concerned not only with health but also with the economy, their work situation, politicians, and the future (Table 6).

Regarding *Health* (the “core” situation), none of the I-COVID participants declared they were “very much” concerned, but most of them stated they were “somewhat” concerned (57.1%) or “rather” concerned (35.7%). Nevertheless, 12.3% of F-COVID, non-infected individuals reported being “very much” concerned with health, and 27.6% and 46.2%, respectively were “rather” or “somewhat” concerned. However, 10.6% reported not being at all concerned with health.

Regarding the *Economy*, widely considered the most important repercussion of the COVID-19 pandemic, 21.4% of individuals in the I-COVID group declared not to be concerned, while in the F-COVID group, 36.9% of individuals reported not being concerned about this topic. Furthermore, while half the participants in the I-COVID group considered themselves to be “somewhat” concerned and 14.3% “rather” concerned, in the F-COVID group 6.3% were “very much concerned”, 14.3% “rather” concerned, and 37.2% “somewhat” concerned.

Regarding *Work*, it must be taken into consideration that most of the participants were retired older adults; thus, 17.9% of F-COVID individuals and 21.4% of I-COVID individuals declared not to be concerned and very few declared to be “very much” concerned. Nevertheless, adding response alternatives, 77.4% of the F-COVID and 71.3% of I-COVID groups felt some concern.

Regarding *Politics*, although 10% of F-COVID individuals and 7.1% of I-COVID individuals declared not to be concerned at all, a high proportion in both groups reported being very much or rather concerned, specifically 68.1% of F-COVID and 50% of I-COVID.

Finally, the *Future* seems to be an important source of concern; thus, only 3% of F-COVID and 7.1% of I-COVID individuals considered themselves not to be concerned with it. Individuals declaring to be “very much” concerned represented 34.9% of F-COVID and 21.4% of I-COVID groups. Adding all categories, 95.7% of F-COVID and 85.6% of I-COVID individuals declared concern about the future to some degree.

In summary, when answering the question, “To what extent are our items considered to be of concern to participants?”, the two subgroups show some differences: the F-COVID group is more concerned with health than the I-COVID group. Taking into consideration the two highest labels—“rather” and “very much”—together, health is the lowest category of concern. The highest aspects of concern are politics and the future in the two groups and, in third place, work. Contrary to our prediction, the lowest categories with the most differences among our two groups were health and the economy. In fact, a summary of this section could be entitled: *From COVID-19 to political crisis and future threat*.

## 4. Ageism in Times of Pandemic?

A pandemic has tremendous repercussions both at the individual level and at the population level. Not only can it cause an increase in mortality or a reduction of life expectancy; it can influence abstract components such as social values or stereotypes. Changing the subject of research also requires changing the methods and procedure of inquiry. The focus of this last section shifts from individuals suffering a situation to a society changing its images and feelings about a group particularly affected by the pandemic: groups of older adults.

As has already occurred in our recent history [15], a pandemic is an extremely dangerous situation not only because there is concern about the future of human life and other negative socio-economic events, as reported in our survey, but because it is a historical occurrence that promotes extremisms, among them ageism. Predictions and warnings in this regard were made public very early on, at the beginning of 2020, by an international group of social scientists [16] demanding the avoidance of ageism and the fostering of intergenerational solidarity and declaring that “with the pandemic there has been a parallel outbreak of ageism” (p. 1). This statement is based on an analysis of public discourse in social media and public announcements made by government representatives emphasizing the extreme frailty of older adults and their cost to society and overgeneralizing some of their traits to the entire older population, which is evidently highly diverse.

Nevertheless, the repercussion from this overgeneralization could have a boomerang effect on society. Thus, following Levy’s embodiment theory [17] positing that “the extension of older adults’ negative stereotypes can be internalized by people of all ages and, when these views become self-relevant, influencing older persons´ beliefs about their own aging, they can detrimentally impact health” (p. 1), as a diversity of studies were supported ([18,19,20] among others). Along similar lines, a recent study assessing negative cultural views about older adults in 29 European countries determined that they are negatively associated with active aging both at the individual and population levels. This also supports the hypothesis that the negative cultural views of older adults could be considered a threat to health at the population and individual levels [21]. Finally, it must be emphasized that the study by Ayalon et al. [16] does not finish with negative predictions based on psychosocial theories, but with recommendations for combating ageism and increasing intergenerational solidarity. The key question here is whether predictions and recommendations coming from social science have had a positive influence on what is going on across the world.

Regarding ageism in Spain during a pandemic, as semantically parallel to sexism, feminism, etc., there is a concept formulated by Butler [22], who defined it using three interconnected components: prejudice (or negative attitudes about this age group), behavioral discrimination against older people (including institutional practices and policies against this age group), and negative images or stereotypes against older people, old age, and the aging process.

During the last 30 years, we have been devoted to assessing age stereotypes (at the individual and population levels) and from 1990 to 2005, we identified a positive profile of aging stereotypes showing an improvement in the views of older adults [23,24]. What was occurring in 2020 during the pandemic regarding ageism? In spite of the fact that ageism (“*edadismo*” in Spanish) is a recently adopted Anglicism in Spanish (across Spain and Latin American), Google provided only 79,500 results, while 3,060,000 results appeared with an English search. Consistent with a similar study by Ayalon et al. [21], three sources of information about ageism in Spain were used: (1) data from a newspaper, (2) two surveys about negative attitudes developed in Spain, and (3) information coming from the new technology of social networks.

1. Bravo-Segal and Villar [25], examined verbal statements and representations referring to older adults in headlines in the two national newspapers with the highest circulation (ABC and El País), as authors stated, “during the most critical phase of the pandemic in Spain” (p. 266). Results reported: “71.4% of headlines representing older adults unfavorably presented them as a homogenous group and associated them with death, deficiencies in residential care, or extreme vulnerability. The presence of certain potentially derogatory or improper terms (paternalistic terms such as “elderly” or “grandparents”) was consistent with those negative representations”.
2. García-Soler, Castejón, Marsillas, Del Barrio, Thompson, and Díaz-Veiga [26] developed an empirical study about ageism based on three assumptions: (a) Older adults are the most affected by COVID-19 and Spain is also one of the countries that has suffered the greatest impact in nursing homes; (b) In the media and in society in general, COVID-19 is being considered as a problem of older people, falling into ageist approaches on numerous occasions; (c) Finally, they considered that the problem of ageism in society is not well known in relation to the health crisis caused by the Covid-19 pandemic. Based on these assumptions, (d) 840 participants responded to an online questionnaire containing items about social inequality, and opinions and attitudes about older people during the COVID-19 pandemic in Spain, and (e) the authors reached the following conclusions: (i) there were stereotypes and discriminatory attitudes, especially in younger groups, and described what several authors understood as intergenerational tension; (ii) the authors concluded with a prediction—perhaps premature—that these stereotypes can be converted to cultural images and thus have serious effects, transforming into policies which affect basic rights, especially autonomy, access to resources, and dignity.
3. Giving voice to older adults through the Older Adults Report issued by the UDP (Unión Democrática de Pensionistas)—BARÓMETRO UDP AÑO VIII No. 1: June of 2020. Ref.: 20065-20133 (I) *N* = 454 individuals older than 65 years, a representative sample by habitat and region (error ≤ 5%), were surveyed. The following results were obtained: (1) 55.2% considered that older adults suffered discrimination due to their age during the COVID-19 crisis, but only 10.9% felt that they were discriminated against; (2) 69.4% stated that they were treated appropriately by most entities (health services, citizens, NGOs, etc.) except for politicians, which is also a concern in our sample; (3) Among the 10.9% feeling discrimination, 61.6% reported being discriminated against when accessing specialized medical services. This opinion is more generalized among individuals within the 65–74 years age range with university studies and living with others.
4. Social networks could be a new source of information for the triangulation of ageism (at least when it is amplified as reported by JAGS), which seems to be penetrating the scientific literature and can be exemplified by the “BoomerRemover” Twitter hashtag. Thus, researchers from Mexico [4] collected a total of 18,128 tweets and selected and analyzed a random sample of 351 (91.7% belonged to individuals). The most common types of tweets were personal opinions, informative tweets, jokes/ridicule, and personal accounts. Overall, 72 tweets (21.9%) likely intended to ridicule or offend someone and 21.1% had content implying that the life of older adults was less valuable or downplayed the relevance of COVID-19. They concluded that almost one-quarter of the tweets analyzed had ageist or potentially offensive content toward older adults.

In summary, after reviewing several studies from different sources, ageism seems to be increasing in Spain as the COVID-19 pandemic continues. As Levy pointed out—and was cited at the beginning of this section—in addition to the consequences of the pandemic, such as the economic crisis and political instability (among others), we must be aware that ageism could have a boomerang effect on our own society. Nevertheless, much more research must be conducted in order to verify the extent to which ageism is a local issue or, as has already been claimed, is being expressed internationally. Perhaps the only solution is to combat ageism by promoting and enlarging intergenerational solidarity.

## 5. Conclusions

After analyzing the responses from 315 older adults (sample divided into the two subsamples—COVID-19 Free (*N* = 301) and COVID-19 Infected (*N* = 14) to an online questionnaire containing 50 items divided into 8 sections, our conclusions are the following:

1. COVID-19 experiences and protective and risk factors. Although the Disease-Free (F-COVID) and Infected (I-COVID) participants did not differ in their information sources and adhered to lockdown, I-COVID individuals seemed to be infected by being in touch with other COVID-19 patients. Knowledge of COVID-19 among the participants of this study was generally good. This could be due to the high level of education of respondents.
2. Treatment and care received by I-COVID individuals. From a statistical point of view, it must be concluded that most I-COVID individuals were satisfied with the care received. Some of them reported diversity mainly in primary health care at the beginning of the pandemic when the health service collapsed due to the unforeseen situation.
3. Self-reported health during the pandemic. Most of the participants in both groups—COVID-19-free and COVID-19-infected—rated themselves with “very good” health, and very few of them rated themselves with “worse health” compared to others of the same age and gender. Only when they were asked to compare their health before and after the pandemic did differences among them appear, and F-COVID self-valuated better than I-COVID. In fact, in order to explain their very good subjective health, two hypotheses arise: I-COVID subjects suffered COVID-19 at the beginning of the pandemic or they suffered it in a very mild form.
4. Changes in healthy lifestyles. As expected, while physical exercise was reduced in both F-COVID and I-COVID, cognitive activity, healthy diet, and drug prescription fulfillment were maintained in both.
5. Unhealthy behaviors as a response to the COVID pandemic. Results showed that two unhealthy behaviors, specifically overeating and drinking alcohol, occurred in at least half of both F-COVID and I-COVID individuals as a response to the pandemic. Moreover, associations were found between unhealthy behavior and lifestyle changes.
6. Emotions, coping, and self-efficacy. Both the verbal reports of anxiety and feelings of fear were higher in the I-COVID than in the F-COVID group, but this was also confirmed through more sleeping disturbance in the Infected group compared to the COVID-19-free. In contrast, the I-COVID group reported lower levels of depression and irritation. Experiencing quarantine or isolation increases anxiety, depression, posttraumatic stress, and stress levels in individuals [27] and may thus increase fear of the unknown along with thoughts of becoming infected. As I-COVID individuals had already been infected and undergone viral infection, their levels of depression and irritation might be reduced.
7. Experience of COVID-19 disease severity, coping, and self-efficacy. More non-infected individuals perceived COVID-19 to be a serious disease and said the pandemic was a more difficult, stressful situation than others in the past. At the same time, all reported high self-efficiency in coping with the situation. Perhaps a post-hoc explanatory assumption would be that the F-COVID group perceives COVID-19 as a more severe illness than I-COVID because they already suffered this illness as “mild”. Both attributions are generalized to the pandemic and, therefore, all of them share high self-efficacy for coping.
8. Family and social interactions and satisfaction. Both I-COVID and F-COVID participants reported very few changes in their relationships during lockdown, and some of them improved their relationships. Regarding the support received and feelings of satisfaction, the pattern of change is quite similar, with most reporting no changes and even some improvements. Finally, the highest source of improvement in support and satisfaction were children and friends.
9. Concerns about health, work, the economy, politics, and the future. When replying to the question regarding the extent to which the items were considered to be of concern to participants, the two subgroups had some differences: F-COVID was more concerned with health than I-COVID (the group infected by COVID-19). Taking into consideration the two most frequent labels, “rather” and “very much”, together, health is the category of lowest concern. The highest aspects of concern are politics and the future in the two groups and, in third place, work. Contrary to our prediction, the lowest categories with more differences among the two groups were health and the economy. In fact, a summary of this section could be entitled: From COVID-19 to political crisis and future threat.
10. Ageism in times of pandemic? After reviewing several studies from different sources, ageism in Spain seems to be growing as the COVID-19 pandemic continues. We are developing a MOOC course combating ageism (ENCAGEn-CM) Nevertheless, much more research must be conducted because, as is expressed in this survey and others conducted by several institutions and scientists, extreme political influence, as well as the negative perception of politics and politicians, seems to be embedded in other scientific considerations.

This study clearly has many limitations. First of all, our sample is not random—the individuals were volunteers and the administrative procedure was online. Secondly, all information came from self-reporting without any objective data about the I-COVID group. Nevertheless, COVID-19 has a very wide variety of levels of severity and it is likely our 14 COVID-infected individuals reported true information in their health report. Third, we know that impression management is a very common source of error of self-report, and perhaps a control for it should have been included. Fourth, new COVID-19 pandemic waves give us the opportunity to replicate our current results. Finally, it is necessary to continue researching agism as a potential consequence of the COVID-19 pandemic as well as working to denounce and fight against it.

## Figures and Tables

**Table 1:** Sociodemographics.

Subsamples	*N* = 301 (COVID-19-Free)	*N* = 14 (COVID-19-Infected)
	**Mean, Range**	**Mean, Range**
**Age**	Mean age: 70.63 yrs. Range: 60–93 yrs.	Mean age: 65.79 yrs. Range: 60–77 yrs.
	*N* (%)	*N* (%)
**Sex**		
Male	158 (53%)	6 (42.85%)
Female	143 (47%)	8 (57.14%)
**Marital Status**		
Married/cohabiting	221 (73.4%)	10 (71.4%)
Divorced	31 (10.3%)	1 (7.1%)
Single	11 (3.7%)	0
Widow/er	37 (12.3%)	3 (21.4%)

**Table 2:** Perceived health compared to that before the pandemic.

Perceived Health	*N* = 301 COVID-FREE	*N* = 14 COVID-INFECTED
	*N* (%)	*N* (%)
How would you describe your health?		
Bad	3 (1%)	0
Somewhat good	8 (6%)	0
Rather good	136 (45.2%)	4 (28.6%)
Very good	144 (47.8%)	10 (71.4%)
Your health compared to individuals of your own age and sex:		
Worse	17 (5.6%)	2 (14.3%)
Same	149 (49.5%)	5 (35.7%)
Better	135 (44.9%)	7 (50%)
How would you rate your health compared to before the COVID19 Pandemic?		
Much worse	4 (1.3%)	0
Somewhat worse	79 (26.2%)	7 (50%)
Somewhat better	188 (62.5%)	7 (50%)
Much better	20 (6.6%)	0

**Table 3:** Healthy lifestyle changes during the COVID-19 pandemic.

Healthy Lifestyles	*N* = 301 COVID-FREE	*N* = 14 COVID-INFECTED
	*N* (%)	*N* (%)
Physical exercise		
Reduced	196 (65.1%)	11 (78.6%)
Maintained	81 (26.9%)	3 (21.4%)
Increased	24 (8%)	0
Cognitive Activity		
Reduced	34 (11.3%)	4 (28.6%)
Maintained	214 (71.1%)	9 (64.3%)
Increased	45 (15%)	1 (7.1%)
Not answered	8 (2.7%)	0
Healthy Diet		
Reduced	12 (4%)	3 (21.4%)
Maintained	242 (80.4%)	11 (78.6%)
Increased	23 (7.6%)	0
Not answered	2 (0.7%)	0
Physicians’ prescriptions		
Reduced	12 (4%)	1 (7.1%)
Maintained	250 (83.1%)	12 (85.7%)
Increased	21 (7%)	1 (7.1%)
Not answered	18 (6%)	0

**Table 4:** Feelings and emotions during the COVID19 pandemic.

Items (1–4)	*N* = 301 COVID-FREE	*N* = 14 COVID-INFECTED
	*N* (%)	*N* (%)
During the COVID-19 pandemic: To what extent did you feel anxious?		
None	71 (23.6%)	0
Somewhat anxious	116 (38.5%)	9 (64.3%)
Rather anxious	89 (29.6%)	4 (28.6%)
Very much anxious	25 (8.3%)	0
During the COVID-19 pandemic: To what extent did you feel depressed?		
None	128 (42.5%)	5 (35.7%)
Somewhat depressed	110 (36.5%)	6 (64.3%)
Rather depressed	50 (16.6%)	0
Very much depressed	13 (4.3%)	0
During the COVID-19 pandemic: To what extent did you feel irritated?		
None	142 (47.2%)	5 (35.7%)
Somewhat irritated	93 (30.9%)	9 (64.3%)
Rather irritated	51 (16.9%)	0
Very much irritated	14 (4.7%)	0
During the COVID-19 pandemic: To what extent did you feel afraid?		
None	71 (26.6%)	3 (21.4%)
Somewhat afraid	131 (43.5%)	4 (28.6%)
Rather afraid	62 (20.6%)	5 (35.7%)
Very much afraid	36 (12%)	2 (14.3%)
Not answered	1	0
During the COVID-19 pandemic: To what extent did your emotions make your daily life activities difficult?		
None	156 (51.8%)	6 (42.9%)
Sometimes	89 (29.6%)	6 (42.9%)
Quite often	45 (15%)	1 (7.1%)
Very much	9 (3%)	1 (7.1%)
Not answered	2 (0.66%)	0
During the COVID-19 pandemic: To what extent did your sleeping patterns change?		
As usual	179 (59.5%)	5 (35.7%)
It takes time to fall asleep	22 (7.3%)	2 (14.3%)
Sometimes I wake up at night	67 (22.3%)	6 (42.9%)
I usually wake up at night	31 (10.3%)	1 (7.1%)

**Table 5:** Changes in family and social interaction, support received, and satisfaction.

		NK/NA (*N* = 14)	Reduced (*N* = 14)	Maintained (*N* = 14)	Improved (*N* = 14)	DK/NA (*N* = 301)	Reduced (*N* = 301)	Maintained (*N* = 301)	Improved (*N* = 301)
		*N* (%)	*N* (%)	*N* (%)	*N* (%)	*N* (%)	*N* (%)	*N* (%)	*N* (%)
Changes in relationships	Partner	2 (14.3%)	0	9 (64.3%)	3 (21.4%)	7 (24.2%)	8 (2.7%)	165 (54.8%)	55 (18.3%)
	Children	0	1 (7.1%)	11 (78.6%)	2 (14.3%)	28 (9.3%)	13 (4.3%)	162 (53.8%)	98 (32.6%)
	Family members	1 (7.1%)	2 (14.3%)	8 (57.1%)	3 (21.4%)	17 (5.6%)	15 (5%)	150 (49.8%)	119 (39.5%)
	Friends	0	1 (7.1%)	7 (50%)	6 (42.9%)	8 (2.7%)	16 (5.3%)	151 (50.2%)	126 (41.9%)
	Others	0	1 (7.1%)	4 (28.6%)	1 (7.1%)	185 (61.5%)	8 (2.7%)	63 (20.9%)	45 (15%)
Changes in support received	Partners	2 (14.3%)	0	8 (57.1%)	4 (28.6%)	76 (25.2%)	4 (1.3%)	151 (50.2%)	70 (23.3%)
	Children	0	0	9 (64.3%)	5 (35.7%)	31 (10.3%)	5 (1.7%)	133 (44.2%)	132 (43.9%)
	Family members	0	0	8 (57.1%)	6 (42.9%)	24 (8%)	8 (2.7%)	158 (52.5%)	111 (36.9%)
	Friends	0	0	6 (42.9%)	8 (57.1%	16 (5.4%)	9 (3%)	151 (50.2%)	125 (41.5%)
	Others	7 (50%)	0	4 (28.6%)	3 (21.4%	162 (53.9%)	4 (1.3%)	83 (27.6%)	52 (17.3%)
	SATISFACTION	DK/NA *N* = 14	No/Few *N* = 14	Rather *N* = 14	Very *N* = 14	DK/NA *N* = 301	No/Few *N* = 301	Rather *N* = 301	Very *N* = 301
		*N* (%)	*N* (%)	*N* (%)	*N* (%)	*N* (%)	*N* (%)	*N* (%)	*N* (%)
Satisfaction with support received	Partner	4 (28.6%)	1 (7.1%)	4 (28.6%)	5 (35.7%)	76 (24.2%)	40 (13.3%)	67 (22.3%)	118 (39.2%)
	Children	0	1 (7.1%)	5 (35.7%)	8 (57.1%)	30 (10%)	38 (12.6%)	82 (27.2%)	151 (50.2%)
	Family members	1 (7.1%)	2 (14.3%)	6 (42.9%)	5 (35.7%)	23 (7.7%)	61 (20.3%)	117 (38.9%)	100 (33.2%)
	Friends	1 (7.1%)	2 (14.3%)	6 (42.9%)	5 (35.7%)	17 (5.7%)	56 (18.7%)	121 (40.2%)	107 (35.5%)
	Social net.	4 (28.6%)	3 (21.4%)	5 (35.7%)	2 (14.3%)	82 (27.2%)	98 (52.3%)	87 (28.9%)	34 (11.3%)
	Others	9 (64.3%)	1 (7.1%)	2 (14.3%)	2 (14.3%)	208 (69.1%)	40 (13.3%)	36 (12%)	17 (5.6%)

**Table 6:** Concerns about Health, Work, and the Economy.

	*N* = 301 COVID19-FREE (CF)	*N* = 14 COVID19-INFECTED (CI)
	*N* (%)	*N* (%)
Health		
No concerns	32 (10.6%)	0
Somewhat concerned	139 (46.2%)	(57.1%)
Rather concerned	83 (27.6%)	5 (35.7%)
Very much concerned	37 (12.3%)	0
No answer	10 (3.3%)	1 (7.1%)
Economic		
No concerns	111 (36.9%)	3 (21.4%)
Somewhat concerned	112 (37.2%)	7 (50%)
Rather concerned	43 (14.3%)	2 (14.3%)
Very much concerned	19 (6.3%)	0
No answer	16 (5.3%)	2 (14.3%)
Work		
No concerns	54 (17.9%)	3 (21.4%)
Somewhat concerned	94 (31.2%)	2 (14.3%)
Rather concerned	101 (33.6%)	7 (50%)
Very much concerned	38 (12.6%)	1 (7.1%)
No answer	14 (4.7%)	1 (7.1%)
Politics		
No concerns	30 (10%)	1 (7.1%)
Somewhat concerned	57 (18.9%)	5 (35.7%)
Rather concerned	107 (35.5%)	4 (28.6%)
Very much concerned	98 (32.6%)	3 (21.4%)
No answer	9 (3%)	1 (7.1%)
The future		
No concerns	9 (3%)	1 (7.1%)
Somewhat concerned	45 (15%)	1 (7.1%)
Rather concerned	138 (45.8%)	8 (57.1%)
Very much concerned	105 (34.9%)	3 (21.4%)
No answer	4 (1.3%)	1 (7.1%)

## Data Availability

Data supporting reported results can be requested from the authors.
